# Post-Vaccination Detection of SARS-CoV-2 Antibody Response with Magnetic Nanoparticle-Based Electrochemical Biosensor System

**DOI:** 10.3390/bios13090851

**Published:** 2023-08-26

**Authors:** Duygu Harmanci, Simge Balaban Hanoglu, Gozde Akkus Kayali, Evrim Durgunlu, Nursima Ucar, Candan Cicek, Suna Timur

**Affiliations:** 1 Central Research Test and Analysis Laboratory, Application and Research Center, Ege University, Izmir 35100, Türkiye; suna.timur@ege.edu.tr; 2 Department of Biochemistry, Faculty of Science, Ege University, Izmir 35100, Türkiye; simge93balaban@gmail.com (S.B.H.); evrimdurgunlu@gmail.com (E.D.); nursima889@gmail.com (N.U.); 3 Department of Medical Microbiology, Faculty of Medicine, Ege University, Izmir 35100, Türkiye; gozdeakkus1306@hotmail.com

**Keywords:** SARS-CoV-2, COVID-19 vaccine, immunoassay, biosensor, anti-S antibody, anti-N antibody

## Abstract

Here, we report magnetic nanoparticle-based biosensor platforms for the rapid detection of SARS-CoV-2 antibody responses in human serum. The use of the proposed system enabled the detection of anti-SARS-CoV-2 spike (S) and nucleocapsid (N) proteins at a concentration of ng/mL in both buffer and real serum samples. In particular, the protocol, which is considered an indicator of innate immunity after vaccination or post-infection, could be useful for the evaluation of antibody response. We included a total of 48 volunteers who either had COVID-19 but were not vaccinated or who had COVID-19 and were vaccinated with CoronoVac or Biontech. Briefly, in this study, which was planned as a cohort, serum samples were examined 3, 6, and 12 months from the time the volunteers’ showed symptoms of COVID-19 with respect to antibody response in the proposed system. Anti-S Ab and anti-N Ab were detected with a limit of detection of 0.98 and 0.89 ng/mL, respectively. These data were confirmed with the corresponding commercial an electrochemiluminescence immunoassay (ECLIA) assays. Compared with ECLIA, more stable data were obtained, especially for samples collected over 6 months. After this period, a drop in the antibody responses was observed. Our findings showed that it could be a useful platform for exploring the dynamics of the immune response, and the proposed system has translational use potential for the clinic. In conclusion, the MNP-based biosensor platform proposed in this study, together with its counterparts in previous studies, is a candidate for determining natural immunity and post-vaccination antibody response, as well as reducing the workload of medical personnel and paving the way for screening studies on vaccine efficacy.

## 1. Introduction

A novel coronavirus that first appeared in Wuhan, China, in December 2019, soon dubbed severe acute respiratory syndrome 2 (SARS-CoV-2), triggered the COVID-19 pandemic and had an unprecedented global impact. Knowing that herd immunity takes time, researchers accelerated their efforts to develop a vaccine against COVID-19. As a result of vaccine trials conducted worldwide using various technologies, the first vaccine to be used was CoronoVac or Sinovac. This vaccine was also the first used in Türkiye and is a kind of inactivated vaccine [1]. After this vaccine, the BNT162b2 vaccine (Pfizer-BioNTech), which has been proven to be more effective and is accepted worldwide, was added to the regime in Türkiye [2].

As important as the rapid detection of the disease is at the onset of a pandemic, the humoral immune response after transmission of SARS-CoV-2 or vaccination is also critical [3]. It is not yet known how long antibodies persist after infection or whether the presence of antibodies confers protective immunity. There is a difference between a person’s antibody production when infected in the past or antibody production when vaccinated [4]. For this reason, there is a need to address the different responses to various vaccines and to regulate the vaccination schedule for the maintenance of vaccine protection according to antibody titers [5]. Although the CDC does not recommend post-vaccination antibody titer measurement for COVID-19, it is essential for pandemic mitigation [6]. Most of the time, antibody titers are determined by using sensitive and specific methods, such as ELISA in combination with PCR. Later, antibody panels produced by different companies (Roche (Basel, Switzerland), Bio-Rad (Hercules, CA, USA), etc.) were added to these tests to provide faster results. Testing each individual prior to vaccination places an enormous burden on the health care system in all countries. A rapid and sensitive test that can be easily administered and reveal an individual’s antibody response to infection or vaccination would facilitate the development of a more effective vaccination strategy. Unfortunately, lateral flow tests, which facilitate disease detection, are at a disadvantage in detecting antibody response. Because an antibody response requires a rapid and quantitative response, LFAs are currently inadequate [7]. Innovative and sensitive systems are needed to detect and monitor the antibody response. Biosensor systems specifically designed for disease detection can meet this need.

A simple biosensor is an analytical device consisting of a bio-receptor, a transducer, and a signal processor. The purpose of these devices is to detect biological substances or monitor biological interactions [8,9]. Transduction methods for biosensor applications are mainly based on the optical, physical, chemical, electrochemical, or mechanical properties of functional bio detection materials (enzymes, antibodies, aptamers, and DNAzyme, etc.) [10,11]. The potential for cost reduction, portability, miniaturization, and integration into point-of-care testing are some of the reasons why electrochemical biosensor platforms are used and preferred as diagnostic tools. The use of various nanomaterials (carbon nanotubes, silica nanoparticles (NPs), organic NPs, metal oxide NPs, etc.) is also common to increase the detection capability of these systems [12,13]. One of these nanomaterials is magnetic NPs (MNPs). Systems containing MNPs can be useful for both diagnostics and antibody response detection. They are among the most interesting in biomedicine because they are easy to synthesize. MNPs have exceptional properties, such as high field irreversibility, high saturation field, superparamagnetism, and additional anisotropy contributions. Their magnetic properties enable them to form a one-way sensing surface and increase the sensitivity of sensing systems [14,15,16]. Compared to approaches based on gold/silver NPs, MNP-based biosensing enables the quantitative detection of analytes as well as the measurement of reaction kinetics. Another important advantage is the possibility of accelerating protein-antibody reaction kinetics by manipulating functionalized MNPs with external magnetic field gradients [17]. Methods based on MNPs are mostly concerned with selectively cleaving nucleic acids. By simply combining lysis and binding steps, they contribute to rapid isolation by the formation of MNP-nucleic acid complexes. Therefore, due to their contribution to extraction, they can contribute to the speed of methods such as PCR in the early molecular diagnosis of COVID-19 [18,19]. In addition, sensor systems based on MNPs have also been evaluated for COVID-19 detection [20,21]. In addition to direct virus detection, different electrode surfaces or measurement approaches based on the conjugation of functionalized MNPs with associated antibodies have been used in these systems. In the first study conducted by our group, an “all-in-one” electrochemical immunosensing platform was proposed for the indiscriminate detection of SARS-CoV-2 and its variants using a combination of MNPs and an antibody cocktail [22]. Since it was observed that homogeneous and reproducible findings could be obtained in conjugation with MNPs with antibodies, it was also adapted to post-vaccination antibody detection.

The aim of this study was to develop an alternative MNP-based biosensor for the sensitive detection of SARS-CoV-2 total spike and nucleocapsid antibodies produced in human serum and to use this sensor to study the profiles of both antibody types in vaccinated and unvaccinated groups newly infected with SARS-CoV-2.

## 2. Materials and Methods

### 2.1. Chemicals, Reagents, and Apparatus

Potassium chloride, potassium hexacyanoferrate (III), N-(3-dimethylaminopropyl)-N′-ethylcarbodiimide hydrochloride (EDC), N-hydroxysuccinimide (NHS), bovine serum albumin (BSA) (lyophilized powder, ≥96%), 2-(N-morpholino) ethanesulfonic acid (MES), and amine-functionalized iron oxide magnetic nanoparticles (30 nm) were purchased from Sigma Aldrich (Steinheim, Germany). Recombinant SARS-CoV-2, S1 subunit protein receptor binding domain (RBD); recombinant SARS-CoV-2 S2 protein, recombinant SARS-CoV-2 nucleocapsid protein, rabbit anti-SARS-CoV-2 S1 RBD antibody; rabbit anti-SARS-CoV-2 S2 antibody; and rabbit anti-SARS-CoV-2 N-Protein antibody were provided by Raybiotech (Peachtree Corners, GA, USA). All chemicals were used at analytical grade purity. Also, phosphate-buffer saline (PBS buffer) (1×, pH 7.4) was used as a working buffer in all experimental studies. Elecsys^®^ Anti-SARS-CoV-2 S and Elecsys Anti-SARS-CoV-2 kits were provided by Roche Diagnostics GmbH, (Rotkreuz, Switzerland).

All electrochemical measurements (differential pulse voltammetry (DPV), cyclic voltammetry (CV), and electrochemical impedance spectroscopy (EIS)) were performed with PalmSens 4 potentiostat (Palm Instruments, Houten, The Netherlands). The DPV and CV measurements were performed in the potential range of −0.4 to 0.8 V, while scan rate for the CV measurements were fixed at 50 mV/s. EIS measurements were also performed between 0.02 and 10 Hz at 0.18 V. Screen-printed carbon electrodes (SPCE) (DropSens-DRP-150 (working electrode; carbon, reference electrode; Ag/AgCl and counter electrode; platinum)) were provided by Metrohm (Herisau, Switzerland). For sensor design, magnets (4.0 mm diameter) were purchased from Megahand Information Technologies (Izmir, Türkiye). All electrochemical measurements were conducted in an electrochemical cell containing 1.0 M KCl and 50 mM [K_3_Fe(CN)_6_] in 1× PBS buffer (pH 7.4).

### 2.2. Subjects and Study Design

Forty-eight vaccinated and unvaccinated SARS-CoV-2-infected subjects participated in this study, most of whom were medical professionals from the Faculty of Medicine, Ege University (Izmir, Türkiye). We separated these samples into four groups, each group having n = 12 samples. The 1st group was infected with SARS-CoV-2 and did not vaccinate, 2nd group was infected with SARS-CoV-2 and vaccinated with CoronoVac, 3rd group was vaccinated with both CoronoVac and Pfizer-Biontech, and 4th group was not infected with SARS-CoV-2 but had had two doses of the Biontech vaccine. Before the subjects were recruited for this study, which was planned as a cohort study, the study was approved by the Medical Research Ethics Committee of Ege University Faculty of Medicine (21-2T/46, 4 February 2021) and informed consent was obtained from all participants. Vaccines administered to the vaccinated group were CoronoVac and Pfizer-Biontech. The unvaccinated group comprised 12 individuals who had COVID-19 but had not received the SARS-CoV-2 vaccine.

Follow-up began from the time at which subjects enrolled in the study showed symptoms of COVID-19 or the PCR test was positive, and blood samples were collected at 3, 6, and 12 months.

In addition, negative (was not infected with SARS-CoV-2 and not vaccinated n = 12) and positive (sample taken during SARS-CoV-2 infection, n = 12) serum samples were collected and applied to control the developed sensor system.

### 2.3. Sample Collection

Blood samples were collected from the Department of Infectious Diseases and Clinical Microbiology, Faculty of Medicine, Ege University Hospital (Izmir, Türkiye). Immediately after sampling, the blood samples were incubated at room temperature for two hours and then serum samples were collected by centrifugation (10 min, 3500 rpm), then stored at −80 °C until use.

### 2.4. Biosensor Design

For the biosensor design, bioconjugation between the SARS-CoV-2 specific S and N proteins to amino-functionalized MNPs was performed according to the study by Singh et al. [23]. Briefly, MNP (0.5 mg), EDC (10 mg), and NHS (1.7 mg) were dissolved in 150 µL MES buffer (0.1 M, pH 6.0) and incubated with shaking for 10 min at room temperature (RT). Using a magnet, the MNPs were collected and washed with distilled water. MNPs were then resuspended with PBS buffer (1×, pH 7.4) and 20 µg/mL S and N protein to a total volume of 300 µL and incubated with shaking for 2 h at ambient conditions. MNP-protein conjugates were collected and washed with 1.0% BSA solution for the hindering of non-specific binding. Finally, the conjugate was resuspended in a 100 µL PBS buffer (1×, pH 7.4).

A screen-printed carbon electrode (SPCE) was used for the biosensor platform design and a neodymium magnet was attached to the back side of the electrode. MNP-protein bioconjugates (20 µL) were immobilized on the electrode surface. Then, different concentrations (1.0, 10, 25, 50, 75, 100, and 200 ng/mL) of SARS-CoV-2-specific anti-spike and (1.0, 2.5, 25, 50, 75, 150, and 200 ng/mL) nucleocapsid antibodies were applied to the surface and incubated at RT. After each modification step, the electrode surface was washed with distilled water and electrochemical measurements (DPV, CV and EIS) were performed.

#### 2.4.1. Optimization Studies

Optimization studies were performed to optimize the concentrations of MNP-conjugated proteins. For this purpose, different concentrations of S and N proteins (10, 20, 50, and 75 µg/mL) and MNPs were conjugated and immobilized on the electrode surface. The current difference resulting from the addition of the relevant antibody at a certain concentration (50 ng/mL) was calculated. In addition, interaction time of antibodies was optimized and DPV measurements were performed at different incubation times (15, 30, 45, and 60 min).

#### 2.4.2. Sample Application

The collected clinical serum samples (grouped as 1st (n = 12), 2nd (n = 12), 3rd (n = 12), and 4th (n = 12), positive serum (n = 12) and negative serum (n = 12)) were undiluted, diluted 10-fold, and diluted 100-fold with 1× PBS buffer. Subsequently, the samples were applied to the functionalizing surface as SPCE/MNP/EDC:NHS/S, or N protein and electrochemical measurements were performed.

### 2.5. Confirmation Analysis: Immunoassay

The evaluation of anti-SARS-CoV-2 S and N levels in the serum samples collected from vaccinated and unvaccinated COVID-19 patients was performed by the quantitative Elecsys Anti-SARS-CoV-2 Kit (09289267190 and 09203079190; Roche, United States). Both tests are intended as an aid in the determination of the immune reaction to SARS-CoV-2. The test procedure was carried out according to the manufacturer’s instructions. The assay principle is an electrochemiluminescence immunoassay (ECLIA). The Elecsys Anti-SARS-CoV-2 immunoassay (Roche Diagnostics International Ltd., Rotkreuz, Switzerland) kit used for validation and comparison in the study was developed by Roche to provide an accurate and reliable method for the detection of antibodies to SARS-CoV-2, to facilitate population screening with high specificity, and to identify past infection status as a potential correlate for subsequent immunity. The kit is dependent on the Cobas immunoanalyzer, which automatically calculates the analyte concentration of each sample in U/mL.

### 2.6. Statistics

Results are presented as mean ± standard mean error (SEM). The data for the calibration were fitting of a linear curve. The limit of detection (LOD) and other analytical features such as sensitivity, specificity, and accuracy were calculated according to the results. The data analysis was performed using GraphPad Prism V8.0 software.

Statistical analyses were carried out using one-way analysis of variance (ANOVA), multiple comparisons were performed using a non-parametric test with Kruskal–Wallis and Dunn’s post-hoc tests, two group comparisons were carried out with a Man–Whitney U test, and correlations between groups were determined with Spearman’s rank test (GraphPad Prism 8.0). *p*-values < 0.05 were considered as statistically significant.

## 3. Results

Vaccine development studies were carried out from the first day of COVID-19. It is possible to examine the developed vaccines under four main groups according to their mechanisms: nucleic-acid-based messenger RNA (mRNA) vaccines (BNT16b2, mRNA-1273, CVnCoV), viral vector vaccines (AZD1222, Sputnik V, Sputnik V Light, Ad5-nCoV (Convidecia), Ad26.COV2.S), inactivated vaccines (NVX-COV2373, CoronaVac, BBIBP-CorV, Wuhan Sinopharm, Covaxin, QazVac, KoviVac, COVIran Berekat), and protein-based vaccines (EpiVacCorona, ZF2001, Abdala) [24]. The target of choice for SARS-CoV-2 is the spike (S) glycoprotein. Inactivated vaccines are vaccines containing inactivated SARS-CoV-2 virus. A target gene is selected for immunogenic activity in mRNA vaccines. In an mRNA vaccine, when encoding the target antigen, cells translate the mRNA into protein in situ [25]. For the detection of both these vaccines and the antibody response to the disease, the separate detection of SARS-CoV-2 S and N antibodies is used in routine clinical applications. For their one-step, rapid, quantitative detection, MNP-based sensor systems that can take advantage of their extraordinary magnetization effects could be useful. Both MNPs and the preferred SPCE electrodes can significantly increase detection sensitivity.

**Scheme 1:**
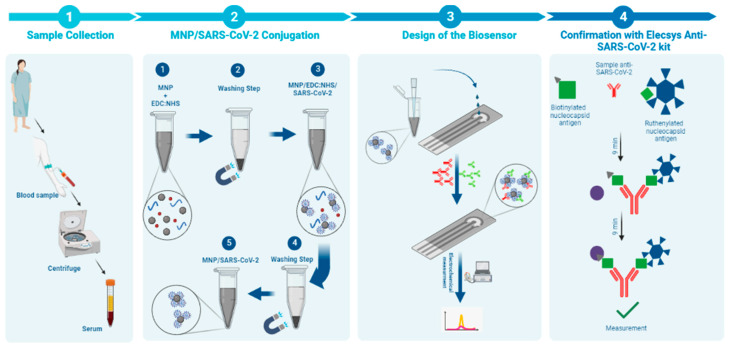
Illustration of the MNP-based electrochemical immunosensor platform for detecting SARS-CoV-2 antibody titers after vaccination. (**1**) Blood samples were collected from volunteers, and their serums obtained by centrifugation. (**2**) Conjugation steps of the MNPs for sample preparation. (**3**) Design and application of the electrochemical sensor in the detection of SARS-CoV-2 antibody titers: S and N antibodies. (**4**) Confirmation of biosensor results with commercial Elecsys Anti-SARS-CoV-2 kit.

In this context, a magnetic-nanoparticle-based biosensor system previously designed for COVID-19 diagnosis [22] was modified in this study to test anti-S and -N antibody levels in vaccinated and unvaccinated groups. In the system, amino-functionalized-MNPs were conjugated with S and N proteins and these conjugates were applied to the surface of SPCE. This electrochemical biosensor system, which enables the detection of antibody levels in serum samples, may be an alternative to commercial ECLIA due to its features such as not requiring large device infrastructure, allowing evaluation without a dilution factor in evaluations after the sixth month, and being miniaturized. The experimental steps carried out in this study are summarized in Scheme 1.

### 3.1. Surface Characterization

**Figure 1:**
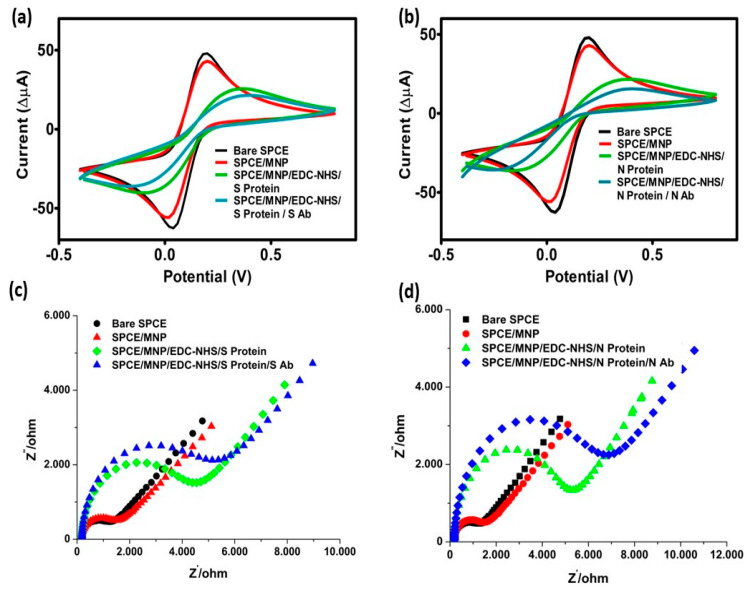
CV and EIS results: (**a**) CV results of biosensor for detection spike (S) Ab; (**b**) CV results of biosensor for detection nucleocapsid (N) Ab; (**c**) EIS results of biosensor for detection spike (S) Ab; (**d**) EIS results of biosensor for detection spike (S) Ab.

The surface modification of the biosensor was investigated with electrochemical measurements (CV and EIS) (Figure 1). When amino-functional MNP and MNP conjugated to protein (S or N protein) fall on bare SPCE, bulky groups are formed on the electrode surface. SEM images were generated to characterized the MNPs and conjugates (Figure S1). The groups hindered electron transfer to the surface and the peaks were decreased, as shown in CV results (Figure 1a,b). When antibodies (S or N Ab) were applied to the electrode in the next step, the electrochemical signal behavior was similar and decreased. According to these results, it can be said that electrode surface modification and protein-antibody interaction were successfully carried out. In addition to CV, electron transfer resistance measurements were performed using EIS to verify the success of the surface modification. The results were analyzed using the Nyquist plot. The Nyquist diagram consists of two parts, with the lower frequencies showing the redox behavior on the electrode surface and the upper frequencies with semicircular peaks showing the expressive electron transfer resistance. Similar to CV, EIS peaks grew after each modification step (Figure 1c,d). This status proves the successful surface modification. (Also, data of CV and EIS can be found in Table S1.)

### 3.2. Optimization Results

**Figure 2:**
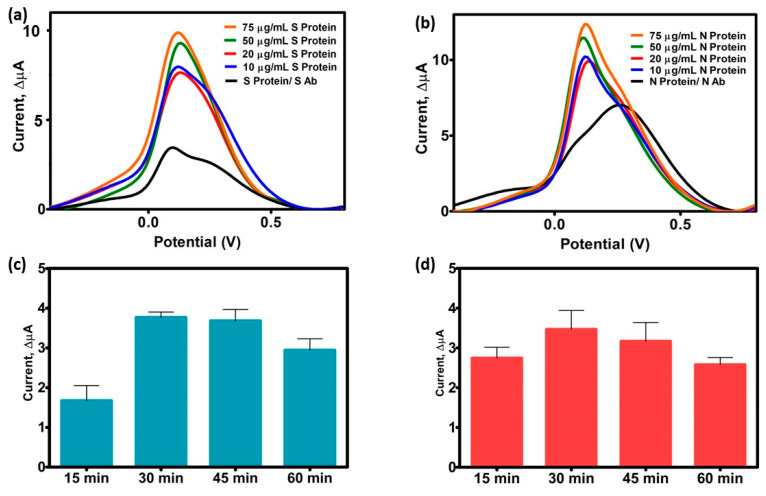
Optimization results for (**a**) S protein concentration, (**b**) N protein concentration, (**c**) interaction time with S protein to S Ab, (**d**) interaction time with N protein to N Ab.

In order to optimize the designed sensor system, the different concentrations of S and N proteins were conjugated with MNP. The same concentration of S and N Ab was added to the modified electrode surface with each conjugated MNP protein. DPV measurements were performed and different currents were calculated. The best current drop in DPV measurements for conjugation of MNP proteins conjugation was found at 20 µg/mL. As shown in Figure 2a,b, 20 µg/mL was found to be the optimum protein concentration for both proteins.

Another optimization parameter was the incubation time of the interaction between MNP protein conjugations and antibodies. After the addition of N- and S-Ab to the MNP-protein conjugations, DPV measurements were performed at different incubation times. As shown in Figure 2c,d, optimum incubation times were determined as 30 min.

### 3.3. Analytical Parameters

**Figure 3:**
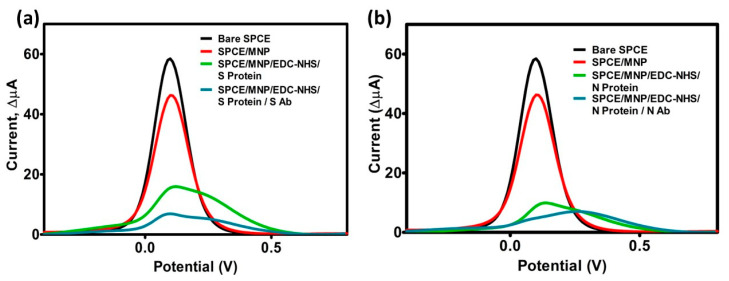
DPV results: (**a**) biosensor for detection spike Ab, (**b**) biosensor for detection N Ab.

To determine the analytical parameters, DPV results were used. DPV peaks decreased after each modification step, consistent with results from CV and EIS (Figure 3).

**Figure 4:**
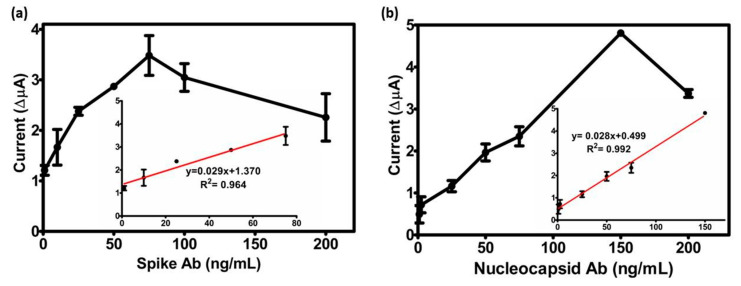
Calibration graphs of the developed biosensor systems. (**a**) Measurement for MNP/EDC-NHS/S protein/S Ab. Calibration curve was shown in the inset; (**b**) Measurement for MNP/EDC-NHS/N Protein/N Ab. Calibration curve was shown in the inset.

A calibration chart was created based on the DPV results. The graphics were created by using the current difference of the peaks obtained after the addition of antibodies with MNP-protein conjugation. (Figure 4). While the linear range for the system prepared with spike antibody was 1.0–75, it was reported to be 1.0–150 ng for the system prepared with nucleocapsid antibody. In both biosensor systems, current differences decreased after the highest point of the linear range at high antibody concentrations. This situation can be explained by the fact that the antibodies had already bound to the proteins on the surface in the highest concentration and the excess antibodies were deposited on the surface. Excess antibodies that accumulated on the surface started to prevent the diffusion of the redox mediator to the electrode surface and the current decreased. In addition, analytical parameters such as limit of detection (LOD) and repeatability were calculated for both systems and the results are summarized in Table 1.

LOD values were calculated with the formula [3 × (S.D.)/m (slope) value]. LOD values of the developed sensors for detection of S Ab and N Ab were calculated as 0.98 and 0.894 ng/mL, respectively. Repeatability values were determined by the standard deviation (±S.D.) of the current difference resulting from measurement at the same concentration Ab at different electrodes. Furthermore, the developed sensors were shown to have high reproducibility and good analytical parameters with coefficients of variation of less than 10% for both sensors.

### 3.4. Interference Results and Sample Application

**Figure 5:**
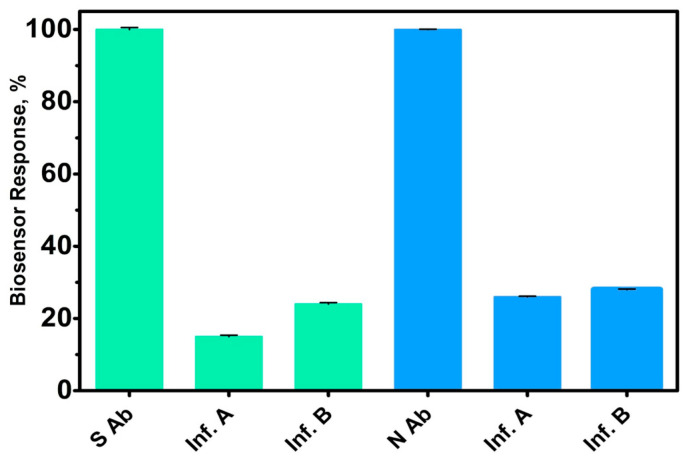
Interference results (green color represents the S-protein modified sensor system and blue represents the N-protein modified sensor system).

Influenza A and B antibodies were used as interference molecules. S and N protein were conjugated with MNP as mentioned above and applied on the electrode surface. Both Abs (25 ng/mL) were applied to surface functionalized with S and N protein. The differences in the currents were compared. For the sensor system prepared with S protein, the biosensor response of influenza A was calculated to be 15%, whereas the response of influenza B was determined to be 24%. Moreover, the biosensor response of influenza A was calculated to be 26%, whereas the response of influenza B was determined to be 28% for the sensor system prepared with N protein. Sensor system responses to Inf A and B were less than 30% (Figure 5). This indicates that the platforms are specific to SARS-CoV-2 Ab.

**Figure 6:**
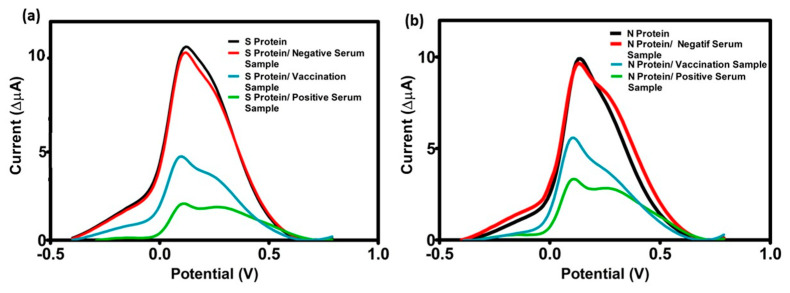
DPV results for positive and negative serum samples with (**a**) S−protein modified sensor system and (**b**) N−protein modified sensor system.

The level of antibodies formed in the body as a result of vaccination studies and the level of determination of antibodies formed in the body in the case of COVID-19-positive results were investigated using the sensor systems developed in this study. Furthermore, negative patient serums were applied to the sensor systems, and the results obtained were compared. As shown in Figure 6a,b, no significant difference was observed in the measurements with negative serum samples for both sensor systems, whereas a large decrease was observed in the measurements with serum samples from COVID-19-positive individuals.

**Figure 7:**
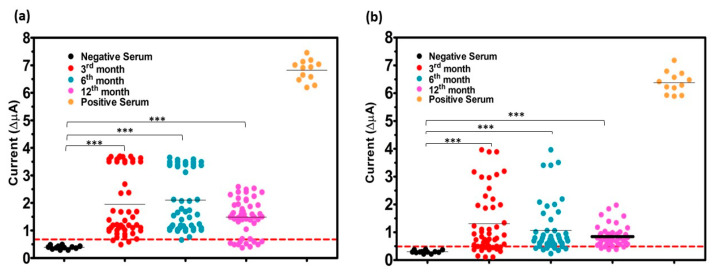
Electrochemical signals results for negative, COVID-19 patients’, vaccination, and post-infection serum samples (the red lines represent the threshold lines). (**a**) S-protein-modified sensor system; (**b**) N-protein modified sensor system. Multiple comparisons were performed using a non-parametric test with Kruskal–Wallis and Dunn’s post-hoc test. [***: *p* < 0.001 compared with the negative group].

Statistical analyses were performed between patients who participated in different vaccination studies, COVID-19-positive patients, and negative serum samples. For the S-protein-modified and N-modified sensor systems, there is a significant difference between participation in the vaccination studies at months 3, 6, and 12 and negative serum samples (*p* < 0.001). As seen in Figure 6 and Figure 7, the current variations obtained from positive samples are quite high and are outside the measurement ranges of the sensor systems. Statistical results are similar for the between participation in the vaccination studies and positive serum samples (*p* < 0.001). In this case, it can be said that the sensor systems are only suitable for the detection of antibodies induced by vaccination. All current differences are listed in Table S1.

### 3.5. Confirmation and Comparison Results

**Figure 8:**
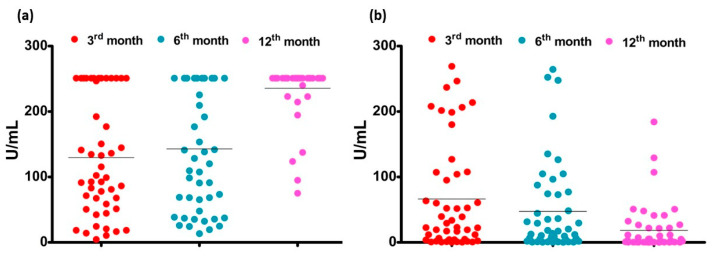
Confirmation studies using Elecsys Anti-SARS-CoV-2 kits for Anti-SARS-CoV-2 S and N Abs. (**a**) Anti-S Ab levels (U/mL); (**b**) Anti-N Ab levels (U/mL).

To confirm the diagnostic accuracy of the developed sensor, we performed a routine test of the quantitative Elecsys Anti-SARS-CoV-2 Kit (09289267190 and 09203079190; Roche, United States). The assay principle is an electro chemiluminescence immunoassay (ECLIA) for the detection of anti-S and -N antibody levels of all groups. After the completion of the tests, we compared the results collected from developed sensor platforms with those of the ECLIA (Figure 8). Accordingly, the highest anti-S and -N levels were observed at the 6th month and started to decrease after the 6th month.

**Figure 9:**
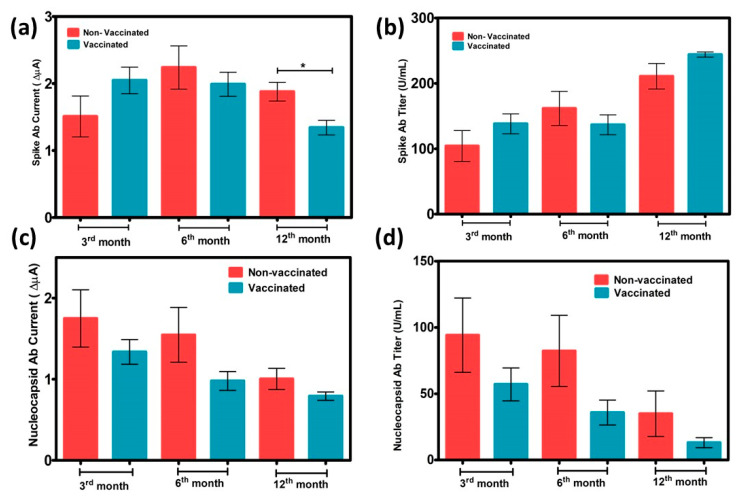
Comparison of anti-S and anti-N levels of the findings obtained by both methods according to months. (**a**) Comparison of anti-S levels obtained with the developed biosensor platform for different months. (**b**) Comparison of anti-S levels obtained with the ECLIA for different months. (**c**) Comparison of anti-N levels obtained with the developed biosensor platform for different months. (**d**) Comparison of anti-N levels obtained with the ECLIA according to months. The comparison of two groups was followed by Mann–Whitney (two-tailed). * *p* < 0.05.

When the findings obtained from both methods were compared between the vaccinated (2nd, 3rd, and 4th) and unvaccinated (1st) groups according to the months, it was observed that the findings overlapped for the 3rd and 6th months, although variability was observed in the 12th month’s results. We discussed possible reasons for this difference in the discussion section (Figure 9).

Analyses performed to observe the correlation between the current values measured with the developed sensor and the concentration values obtained with ECLIA showed that low current values are associated with high concentrations. In other words, the higher the concentration, the higher the current difference value obtained as the current decreases. Therefore, there is a strong positive correlation between both methods, as obtained from the findings, especially for the 3rd and 6th months. At month 12, the findings are overestimated for ECLIA due to a correction factor applied as recommended by the manufacturer. In contrast, no correction is applied in the biosensor system, so current differences are given directly.

## 4. Discussion

The importance of assessing the antibody response to infection or vaccination has received renewed attention with the COVID-19 pandemic. This is because the response to infection or vaccination increases over time, but then decreases. The antibody response is relevant not only for protecting an individual’s own health but also for public health and health system burden. Therefore, we propose an MNP-based biosensor system in this study. The system, which was previously developed using swap samples for the detection of COVID-19, was modified to make it suitable for antibody response detection [20]. The main principle of this MNP-based biosensor functioning is to recognize anti-S (total) or -N antibodies using S or N proteins to convert it to a quantifiable signal, which is proportional to the analyte concentration in the reaction. The conversion of a bio-recognition event into a quantifiable signal is achieved here by utilizing electrochemical sensing technology.

To ensure specific binding in the developed MNP-based biosensors, amino-functionalized MNPs and EDC-NHS chemistry were employed. We applied dilution to minimize the matrix effect of serum and no surface degradation of SPCE electrodes was observed. We obtained sensitive and reproducible findings. It used each electrode once in these systems, allowing detection at the ng/mL level. Although the biosensor has the advantage of being cleaned and reused, further studies on the reusability of the electrodes are appropriate, as it creates a difference between measurements. Its routine counterpart, ECLIA, was successful in determining the concentration of both antibodies, and although it has a time disadvantage compared to ECLIA, this is the first MNP-based biosensor system proposed for antibody response. Other recently proposed electrochemical, optical, and other sensory systems are summarized in Table 2. Studies on systems for the detection of antibody response after vaccination or because of innate immunity are new and few. To allow comparison, the table includes all other systems rather than only electrochemical systems.

Examining the objectives of existing qualitative tests is an approach to distinguish between natural infection and vaccination-induced immunity, and to monitor the effectiveness of immunity and decide on the need for revaccination. For example, the BNT162b2 vaccine (Pfizer) induces an Ab response only against the S protein, whereas natural infections induce Abs at least against both S and N proteins [28]. Currently, enzyme-linked immunosorbent assay (ELISA) is one of the most common approaches for COVID-19 serology testing, but it has shortcomings such as being labor intensive and requiring large equipment. Biosensor systems are also advantageous because they do not require equipment or expertise.

In intergroup evaluations, it was observed that anti-N levels were higher in the first group in relation to natural immunity. Anti-N levels are known to increase in natural immunity [34]. This elevation gradually decreased, especially after the sixth month. In the fourth group vaccinated only with Biontech, anti-S levels were higher, as predicted [35].

The difference observed between the ECLIA and the developed system findings, especially in the 12th month findings, is actually related to not applying a dilution factor, which is recommended in the ECLIA procedure. In order to ensure consistency between the values compared here, the calculation was made without applying this factor. However, at 12 months, both antibody levels dropped significantly in all groups. This is an important indicator of the need for revaccination. Therefore, the proposed biosensor system provides more sensitive and reliable data.

**Figure 10:**
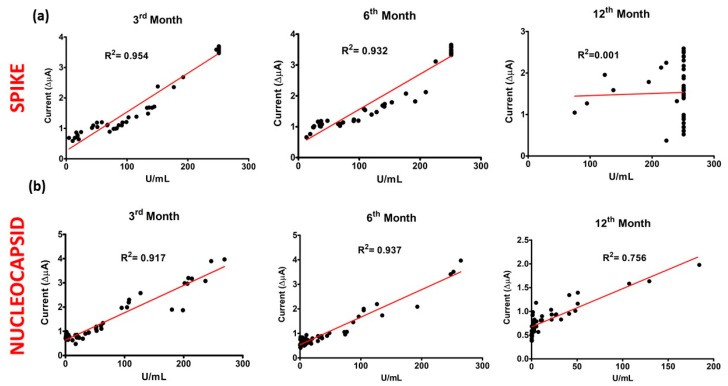
A correlation between ECLIA and proposed biosensor results for the detection of (**a**) anti-S and (**b**) anti-N levels. Correlations between groups were performed using Spearman’s rank test (GraphPad Prism 8.0). *p*-values < 0.05 were considered as statistically significant.

MNP-based biosensor detection results for serum samples of vaccinated and non-vaccinated groups were compared to ECLIA. As shown in Figure 10, the measured biosensor detection ratios for serum samples were highly correlated with ECLIA results. These results demonstrate that the proposed biosensor detection approach provides comparable immunodetection results to established methods.

The entire assay time for performing antibody detection is up to 2 h, which is significantly longer than ECLIA, which completes antibody detection in approximately 20 min. As mentioned earlier, LFA tests are insufficient for immunomonitoring. Therefore, despite the disadvantage of time, this system is an alternative that is easy to use, inexpensive, and does not require infrastructure and experienced specialists for accurate results. One of the most important advantages of the system is its ease of adaptability. In conclusion, the developed MNP-based biosensor system is an alternative sensing platform capable of measuring the post-vaccination status of SARS-CoV-2 S and N antibody levels with high sensitivity. It is a candidate for monitoring the status of COVID-19 vaccination response and the immunity of individuals to COVID-19. Its widespread impact in determining vaccine efficacy and the need for revaccination is clear, not only for the individual but also for reducing the labor-intensive and financial burden on the healthcare system.

## Figures and Tables

**Table 1:** Summary of analytical properties of the proposed sensor.

Analytical Parameters	Values	
	MNP/S Protein/S Ab	MNP/N Protein/ N Ab
Linear Range (ng/mL)	1.0–75	1.0–150
Slope	0.029	0.028
Intercept	1.370	0.499
±S.D. of Slope	0.003	0.001
±S.D. of coefficient	0.139	0.092
Correlation coefficient	0.964	0.992
LOD (ng/mL)	0.978	0.894
Repeatability (±S.D.)	0.04	0.061
Coefficient of variation (%)	2.330	3.460

**Table 2:** Previous electrochemical, optical, and other sensor development studies regarding SARS-CoV-2 antibody response after vaccination.

Sensor Type	Target	Biological Material	LOD	Ref
Nanostructure-coupled nanopore biosensor platform	Neutralizing antibody	Serum	0.2 pM	[26]
Gold micro-interdigitated electrodes biosensor	IgG (anti-S Ab specific)	Serum	AuNp strategy: 200 ng/mLdielectrophoretic (DEP) approach: 2 μg/mL	[27]
Microfluidics-based tool	IgG	Serum	47 ng/mL	[28]
Testing-on-a-probe plus panel	Total antibody (TAb) and surrogate neutralizing antibody (SNAb)	Serum	Various values	[29]
Label-free surface-enhanced Raman scattering biosensing platform	IgG	Tear	1 × 10^−14^ M	[30]
Photonic assay platform	S1 + S2	Serum	4.2 μg/mL	[31]
Fiber optic-biolayer interferometry-based biosensor	Neutralizing antibody (Nabs), anti-receptor binding domain (anti-RBD) BAbs, and anti-extracellular domain of spike protein (anti-S-ECD) BAbs	Serum	Various values	[32]
Grating-coupled fluorescent plasmonics biosensor platform	RBD, spike S1 fragment, spike S1S2 extracellular domain	Serum	Various values	[33]

## Data Availability

All data generated by the study are included in the manuscript or in its Supplementary Material.

## Supplementary Materials

The following supporting information can be downloaded at: https://www.mdpi.com/article/10.3390/bios13090851/s1, Figure S1: SEM images of (a) Amino-functionalized MNP; (b) MNP/EDC-NHS/SARS-CoV-2 S protein (c) MNP/EDC-NHS/SARS-CoV-2 N protein; Table S1: Data of EIS and CV results.
