# Supplementary material for: Post-Vaccination Detection of SARS-CoV-2 Antibody Response with Magnetic Nanoparticle-Based Electrochemical Biosensor System
Source: Biosensors (Basel). 2023 Aug 26;13(9):851. doi: 10.3390/bios13090851 (PMC10526319; doi:10.3390/bios13090851)
Supplement: Supplementary file 1 [file biosensors-13-00851-s001.zip › biosensors-2511069-supplementary.pdf]

biosensors

Supporting Information

Post-Vaccination Detection of SARS-CoV-2 Antibody Response with Magnetic Nanoparticle-Based Electrochemical Biosensor System

Duygu Harmanci, Simge Balaban Hanoglu, Gozde Akkus Kayali, Evrim Durgunlu, Nursima Ucar, Candan Cicek and Suna Timur

Characterization of MNPs

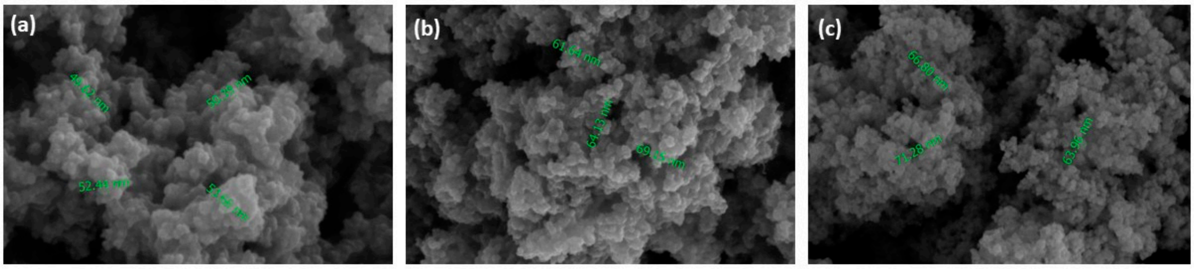

Figure S1. SEM images of (a) Amino-functionalized MNP; (b) MNP/EDC-NHS/SARS-CoV-2 S protein (c) MNP/EDC-NHS/SARS-CoV-2 N protein.

Surface characterization

Table S1. Data of EIS and CV results.

	Cationic Current Value (µA)	Anionic Current Value (µA)	Ohm (Ω)
**Bare SPCE**	-62.611	47.763	983
**SPCE/MNP**	-56.812	43.145	1094
**SPCE/MNP/EDC-NHS/S Protein**	-40.237	25.672	3742
**SPCE/MNP/EDC-NHS/S Protein/ S Ab**	-36.149	21.496	4408
**SPCE/MNP/EDC-NHS/N Protein**	-35.690	21.664	4464
**SPCE/MNP/EDC-NHS/N Protein/ N Ab**	-35.714	15.609	5764
